# The Characteristics of Chinese Orthographic Neighborhood Size Effect for Developing Readers

**DOI:** 10.1371/journal.pone.0046922

**Published:** 2012-10-08

**Authors:** Jing Zhao, Qing-Lin Li, Hong-Yan Bi

**Affiliations:** 1 Key Laboratory of Behavioral Science, Institute of Psychology, Chinese Academy of Sciences, Beijing, China; 2 Graduate University of Chinese Academy of Sciences, Beijing, China; University of Leicester, United Kingdom

## Abstract

Orthographic neighborhood size (N size) effect in Chinese character naming has been studied in adults. In the present study, we aimed to explore the developmental characteristics of Chinese N size effect. One hundred and seventeen students (40 from the 3^rd^ grade with mean age of 9 years; 40 from the 5^th^ grade with mean age of 11 years; 37 from the 7^th^ grade with mean age of 13 years) were recruited in the study. A naming task of Chinese characters was adopted to elucidate N-size- effect development. Reaction times and error rates were recorded. Results showed that children in the 3^rd^ grade named characters from large neighborhoods faster than named those from small neighborhoods, revealing a facilitatory N size effect; the 5^th^ graders showed null N size effect; while the 7^th^ graders showed an inhibitory N size effect, with longer reaction times for the characters from large neighborhoods than for those from small neighborhoods. The change from facilitation to inhibition of neighborhood size effect across grades suggested the transition from broadly tuned to finely tuned lexical representation in reading development, and the possible inhibition from higher frequency neighbors for higher graders.

## Introduction

Once a single word is presented, the readers may mentally activate many other forms, such as words with similar orthography. Coltheart and his colleagues first defined the concept of *orthographic neighborhood* of a target word as all words of the same length that can be generated by changing just one letter while preserving letter positions [1], e.g. orthographic neighbors of “cheat” are “cheap”, “chest”, “cleat”, and “wheat”. The number of these neighbors is called *neighborhood size (N size)*. It has been reported that the orthographic neighbors influenced target word’s processing, and the effects of neighborhood size significantly interacted with task. Facilitatory effects (i.e. faster response for words from large neighborhoods than for those from small neighborhoods) were observed in naming and lexical decision [2]–[5], but these effects disappeared in the semantic categorization task [6], [7] and progressive-demasking task [6], [8]. Many models of lexical processing can give good explanations to the N size effect, such as the dual route model [9], the parallel distributed processing model [10], and the multiple read-out model [11].

Different from alphabetic languages, Chinese as a logographic script consists of block characters without letters, and there should be no orthographic neighborhood according to the definition of Coltheart et al. [1]. However, about 85% of Chinese characters are semantic-phonetic compounds, with the phonetic and semantic radicals informing the character’s pronunciation and meaning to some extent, respectively [12], [13]. By the medium of phonetic radicals, Chinese orthographic neighborhoods can be formed. For instance, characters 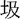 (pronounced/ji1/, meaning *garbage*; the number here refers to tone; the same below), 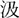 (/ji2/, *draw*), 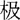 (/ji2/, *unmitigated*), 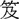 (/ji2/, *book*), 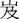 (/ji2/, *danger*), 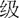 (/ji2/, *class*), 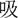 (/xi1/, *absorb*), and 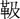 (/sa3/, *shoes*) make up a neighborhood with the same phonetic radical 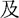 (/ji2/, *and*). The phonetic-radical neighborhood size effect was found in the Chinese adult reading [14], [15]. The result of Bi et al.’s study showed a novel inhibition of neighborhood size in Chinese character naming, in which naming characters from large neighborhoods took longer reaction times and made more errors than naming characters from small neighborhoods [14]. The authors reasoned that because of the low level of phonological consistency in Chinese, in which the consistency is whether a character’s pronunciation agrees with those of its orthographic neighbors [16], [17], the different sounds within a given neighborhood would interfere with the target’s phonological retrieval. This speculation was further confirmed by the following study [15].

For the beginning readers, the orthographic knowledge has not been fully built up, and the orthographic representations are not yet sufficiently specified [18]. The lexical tuning hypothesis [19], [20] proposed that the developing readers were more influenced by the orthographically similar words than skilled readers due to their less finely tuned lexical representation. And the lexical representation would become more finely tuned automatically with the development of reading. According to this hypothesis, greater N size effect was expected in the beginner reading, and this has been proved [21], [22]. Laxon et al. reported that the accuracy in response to words from dense neighborhoods was 10% higher than that in response to words from sparse neighborhoods in lexical decision, naming and spelling tasks for children from the 1^st^ to 3^rd^ grades [22], but this accuracy difference was less than 4% for adults in previous studies [2], [3], [5]. Duñabeitia and Vidal-Abarca found that although all students from the 1^st^ grade to 6^th^ grade showed a significant N size effect, this effect in the 1^st^ graders was the greatest [21]. However, in these studies, the frequency of words was high based on the adult criterion. The frequency of the same word is apparently different between beginning and skilled readers. Previous studies have found that N size effect was more prominent for the lower-frequency words [2]–[4], and then the developmental difference of N size effect was mixed with the word frequency effect.

Moreover, Laxon et al. reported the progressive-gradation development of neighborhood size effect in English words [22], in which the N size effect for the 3^rd^ graders was still greater than the adults. But Duñabeitia and Vidal-Abarca found N size effect was close to the adults early in the 2^nd^ grade in Spanish [21]. Considering that the main difference between Spanish and English is orthographic depth, English is a kind of language with deeper orthography than Spanish, thus it is more difficult for English developing readers to acquire the rules of grapheme-to-phoneme correspondence (GPC). Previous studies revealed that beginning readers preferred to use the lexical route before they acquired the GPC rules [23], [24], and the effect of orthographic neighbors was more prominent in the lexical route than in the GPC route [23], therefore, N size effect develops differently between English and Spanish. It is well known that Chinese belongs to the logographic language system with deeper orthography than alphabetic languages, so it is particularly important to explore the developmental trend of neighborhood size effect in Chinese in order to make clear of the language characteristic’s role in the N-size-effect development. To our knowledge, there has been only one study concerning N size effect in Chinese developing readers [25]. Results of their study showed that an inhibitory N size effect significantly appeared for the 5^th^ graders, especially in the low-consistency condition. However, the error rates for the 5^th^ graders were a little higher (e.g. about 40% in the low-consistency/large-neighborhood condition), and the number of valid stimuli for the 5^th^ graders was only 8 in each condition. Thus, further study was needed to explore the developmental pattern of Chinese neighborhood size effect with more elaborate manipulations.

In the current study, we aimed to explore the development of neighborhood size effect in Chinese character naming among the 3^rd^, 5^th^, and 7^th^ graders, controlling the age of acquisition (AoA) of target characters to match the familiarity of stimuli across different grades. If there is a transition from broadly tuned to finely tuned lexical representation as the lexical tuning hypothesis predicted, beginning readers would show greater N size effect than higher graders. Given that Chinese has relatively deep orthography, it could be expected that developing readers would show a similar N size effect as the adults till higher grades.

## Method

### Participants

One hundred and seventeen students participated in this study, including 40 students from the 3^rd^ grade (20 males, a mean age of 9 years, ranging from 9 to 10 years), 40 students from the 5^th^ grade (21 males, a mean age of 11 years, ranging from 11 to 12 years), and 37 students from the 7^th^ grade (19 males, a mean age of 13 years, ranging from 13 to 14 years). All participants were right-handed Mandarin speakers with normal or corrected-to-normal vision. Informed consents were obtained from their parents as well as their teachers.

### Design and Materials

A mixed 2×3 factorial design was conducted, including a within-subject factor: neighborhood size (large vs. small neighborhoods), and a between-subject factor: grade (the 3^rd^, 5^th^, and 7^th^ grades).

There were thirty-four Chinese characters for each grade. Referring to Li and Kang [26], half of the stimuli belonged to large N size (N size range: 10–23), and the other half belonged to small N size (N size range: 2–7). The number of neighbors significantly differed between the large- and small-N size conditions for each group (Table 1), meanwhile it was balanced across different groups [F(2, 99) = 0.08, p = .92].

**Table 1:** Stimulus characteristics in each condition.

		N size			Consistency			Strokes		
		L	S	t value	L	S	t value	L	S	t value
3^rd^	*Mean*	16.5	4.8	11.0***	0.26	0.25	0.3	9.6	9.0	0.1
	*SD*	4.1	1.5		0.07	0.07		2.2	1.7	
5^th^	*Mean*	15.6	4.9	10.4***	0.28	0.24	1.1	9.9	9.0	1.3
	*SD*	4.0	1.5		0.12	0.13		2.5	2.4	
7^th^	*Mean*	15.4	5.3	8.5***	0.32	0.35	0.8	10.3	10.6	0.9
	*SD*	4.1	2.6		0.13	0.18		2.3	3.2	

Note. 3rd, the third grade, 5th, the fifth grade, 7th the seventh grade. L, large neighborhood size; S, small neighborhood size.

*** p<0.001.

All the characters had left-right structures with the phonetic radicals on the right side, and they had unambiguous meaning, not functional characters. Because the inhibitory effect of Chinese orthographic neighborhood size was prominent in irregular condition (i.e. the target character whose pronunciation is different from that of its phonetic radical) and low-consistency condition [15], [25], meanwhile less than 30% of the semantic-phonetic compounds are regular characters [27], and phonological consistency is much lower in Chinese [28], [29], we selected the irregular characters with a low consistency as target stimuli here. The consistency value (c) was calculated from the relative ratio of the number of orthographic neighbors with the same pronunciation (n) to the whole neighborhood size (N), c = n/N [17]. The consistency and the number of character strokes were controlled between large- and small-N size conditions (Table 1), and there were no group differences neither for consistency [F(2, 99) = 0.02, *p* = .24], nor for the number of strokes [F(2, 99) = 0.88, *p* = .42]. Furthermore, according to previous studies [30]–[32], we controlled AoA of target stimuli by selecting characters from the Chinese books that participants learned in the past two years, so as to control the stimulus familiarity across different grades. Characters used in each grade were showed in Table S1 in the supporting information.

### Procedure

Participants sat before the screen at a viewing distance of 45 cm with a visual angle of 3°. They were asked to read the character loudly when it appeared on the screen. A target character was presented in the center of the screen after a 500-ms fixation cross. The stimulus disappeared upon response, or at the end of a 2000-ms response window. There was a 500-ms fixation cross in the screen between any two adjacent characters. Characters were presented randomly on a DELL laptop, and the experimental procedure was programmed with the E-prime 2.0 software. Reaction times were recorded through a microphone connected to the computer. Before the formal experiment, a practice session including 20 different characters was conducted in order to make the children understand the procedure and avoid the unrelated stimulation to the microphone. In addition, during the experiment, the experimenter sat beside the participants, observing and recording the participants’ response. If the onset of the pronunciation was not the item’s first phoneme, the experimenter would immediately mark this trial.

## Results

The datasets of nine participants were discarded because of error rates higher than 50%. Correct reaction times longer than three standard deviations away from the mean were excluded. Data from the remaining 108 participants (37 students of the 3^rd^ grade, 36 students of the 5^th^ grade, and 35 students of the 7^th^ grade) were put into further analysis.

The mean reaction times (RTs) and error rates (ERs) in each condition were shown in Table 2. A two-way ANOVA for RTs showed that the main effect of neighborhood size was not significant in participant analysis [F_1_(1,105) = 0.65, *p* = .42], and marginally significant in item analysis [F_2_(1,96) = 3.54, *p* = .06]; the main effect of grade was significant in both participant and item analysis [F_1_(2,105) = 3.56, *p*<.05; F_2_(2,96) = 4.92, *p*<.01], post hoc analysis showed that the 3^rd^ graders responded significantly slower than the other two groups (*ps*<.05), and there was no remarkable difference between the 5^th^ and 7^th^ graders (*p*>.1); the two-way interaction was significant in participant analysis [F_1_(2,105) = 8.08, *p*<.01], but non-significant in item analysis [F_2_(2,96) = 0.45, *p* = .64].

**Table 2:** Mean reaction times (RTs) and error rates (ERs) in each condition

Grade		RTs (ms)			ERs		
		L	S	t value	L	S	t value
3^rd^	*Mean*	712.0	739.3	4.3***	0.17	0.20	1.1
	*SD*	123.4	117.3		0.10	0.12	
5^th^	*Mean*	671.0	682.1	1.6	0.17	0.21	0.4
	*SD*	94.2	96.1		0.12	0.12	
7^th^	*Mean*	685.4	660.4	6.8**	0.18	0.17	0.9
	*SD*	107.7	93.9		0.13	0.12	

Note. 3rd, the third grade, 5th, the fifth grade, 7th th seventh grade. L, large neighborhood size; S, small neighborhood size.

*** p<0.001.

** p<0.01.

A paired-sample t-test for the RTs within each group showed a significant N size effect for the 3^rd^ graders in participant analysis [t1 = 4.34, *p*<.001], with shorter reaction times for naming characters from large neighborhoods, while null effect in item analysis [t2 = 1.72, *p* = .10]; a non-significant N size effect for the 5^th^ graders in either participant analysis [t1 = 1.57, *p* = .13] or item analysis [t2 = 1.19, *p* = .24]; while increasing reaction times were found in naming characters from large neighborhoods for the 7^th^ graders in participant analysis [t1 = 6.75, *p*<.01], yet null effect in item analysis [t2 = 0.37, *p* = .71].

The same ANOVA for ERs did not show any significant effects in either participant analysis or item analysis, i.e. neighborhood size effect [F_1_(1,105) = 1.73, *p* = .19; F_2_(1,96) = 0.31, *p* = .58], grade effect [F_1_(2,105) = 0.17, *p* = .84; F_2_(2,96) = 0.25, *p* = .78], and two-way interaction [F_1_(2,105) = 1.73, *p* = .18; F_2_(2,96) = 0.29, *p* = .75].

## Discussion

The present results based on the participant analysis showed a facilitatory N size effect for the 3^rd^ graders (beginning readers), null effect for the 5^th^ graders, and an inhibitory N size effect for the 7^th^ graders (higher graders).

The 3^rd^ graders showed a greater facilitatory N size effect than the 5^th^ graders, this developmental trend was in accordance with the prediction based on the lexical tuning hypothesis, in which greater N size effect should be observed for earlier readers. Moreover, the multiple-route model of orthographic processing could also give another alternative explanation to the current results [33]. According to the model, the influence from orthographic neighbors would increase with the early development of the coarse-grained route, while diminish with the development of the fine-grained route. In the present results, the 5^th^ graders showed a decreased N size effect comparing with the 3^rd^ graders, which was consistent with the hypothesis of the model. As to the developing readers earlier than the 3^rd^ graders, whether there was an increased trend in N-size-effect development is unsolved.

In the present results, the facilitatory effect of neighborhood size for the 3^rd^ graders reflected the contribution of similar orthography. According to the revised Perfetti’s lexical constituency model about the Chinese N size effect in character naming [15], the visual representation of a target character at the feature level activates all characters with the same phonetic radical at the orthographic level. And through the bi-directional connections between the feature and orthographic levels, the activation of orthographic units is strengthened. When there are more characters sharing the same radical, the facilitation will be more prominent.

In contrast with the 3^rd^ graders, the 7^th^ graders showed an inhibitory effect of neighborhood size just like the adults [14], [15]. Li et al. indicated that the inhibitory N size effect resulted from the higher frequency neighbor (HFN), i.e. the neighbor with a higher frequency than the target character [15]. The authors pointed out that, with higher resting activation level, the HFNs would be quickly activated, and become stronger competitors of the target character’s identification. Due to the low level of phonological consistency in Chinese, the neighbor with higher frequency always sounded different from the target character, which interfered with the phonological access of target characters. Moreover, Li et al. also observed a facilitatory effect of neighborhood size when the target character possessed higher frequency than its neighbors, i.e. no existence of higher frequency neighbors [15].

The facilitatory N size effect for 3^rd^ graders was similar with the result in without-HFN condition in Li et al.’s study [15]; while the inhibitory N size effect for the 7^th^ graders was the same as the result in with-HFN condition in Li et al.’s study [15], suggesting a possible HFN effect. However, due to the limitation of stimulus selection for children, we didn’t manipulate the factor of HFN in the experiment. So we reanalyzed the materials of our current study and found 88% of the target characters possessing at least one HFN. Obviously, the 3^rd^ graders weren’t influenced by HFNs in their reading. One possible reason was that character frequencies were calculated according to the adult, and the information of frequency didn’t make sense for 3^rd^ graders. Null N size effect for the 5^th^ graders seemed to be a transitional state indicating the interaction of neighbors’ orthography and HFNs’ phonology. As to the 7^th^ graders, their inhibitory N size effect should be due to the quick activation of HFNs just like the results in the adult naming [15]. With the enhancement of reading ability, the information of lexical frequency for the 7^th^ graders will be more consistent with the adult’s, and the HFNs’ inhibition on target character naming emerged. Previous neuroimaging studies also revealed greater involvement of orthography for beginning readers and greater specialization of phonological processing for skilled readers [34], [35].

As we expected, only high graders (the 7^th^ grade) showed a similar N size effect with the adults. In alphabetic languages, English children after the 3^rd^ grade exhibited a mature N size effect [22], and Spanish children developed closely to the adults in the 2^nd^ grade [21]. Firth indicated that the effect of orthographic neighbors was more prominent in the lexical route than in the GPC route [23]. Then because the orthographic depth of Spanish is shallower than that of English, GPC rules in Spanish would be mastered more easily, and N size effect in Spanish children developed maturely earlier than that in English children. While Chinese lacks of the GPC rules, Chinese developing readers need to make more efforts to establish the association between the visual form of a Chinese character and its sound by the lexical route, in which the lexical retrieval would be greatly affected by orthographically similar representations. Thus, comparing with the alphabetic languages, Chinese children would spend more time on having a similar N size effect with adults.

In brief, the current findings revealed a transition from broadly tuned to finely tuned lexical representation for Chinese developing readers. The facilitatory N size effect in beginning readers suggested a contribution from similar visual forms of orthographic neighbors, and the inhibitory N size effect in higher graders suggested an HFN’s interference with the target character’s access.

It is worth mentioning that all the discussion above was mainly based on the results by participant analysis. In the present study, the results by item analysis showed weak or null effects. So, we should take the current results as suggestive, rather than as conclusive. More studies will be needed in the future to arrive at a clear conclusion.

## Supporting Information

Table S1 **Characters for each grade.**
(DOC)
