# Supplementary material for: The Characteristics of Chinese Orthographic Neighborhood Size Effect for Developing Readers
Source: PLoS One. 2012 Oct 8;7(10):e46922. doi: 10.1371/journal.pone.0046922 (PMC3466200; doi:10.1371/journal.pone.0046922)
Supplement: Table S1 — Characters for each grade. (DOC) [file pone.0046922.s001.doc]

**Supplement**

**Target S1 characters for each grade**

| Grade 3 | | Grade 5 | | Grade 7 | |
| --- | --- | --- | --- | --- | --- |
| Large | Small | Large | Small | Large | Small |
| 读 | 除 | 播 | 坝 | 瞅 | 撑 |
| 缸 | 独 | 沸 | 棒 | 磁 | 炊 |
| 汗 | 话 | 洪 | 拨 | 搓 | 赐 |
| 河 | 活 | 浑 | 衬 | 睹 | 涸 |
| 静 | 猫 | 精 | 促 | 恨 | 恍 |
| 课 | 哪 | 抗 | 钓 | 烘 | 魂 |
| 拉 | 趴 | 裸 | 肺 | 混 | 矿 |
| 洛 | 捧 | 阻 | 减 | 沮 | 滥 |
| 跑 | 秋 | 排 | 括 | 妙 | 腼 |
| 骗 | 晚 | 拼 | 悦 | 呐 | 陌 |
| 抢 | 斜 | 铺 | 俗 | 畔 | 挪 |
| 悄 | 休 | 枪 | 绦 | 偏 | 撇 |
| 纱 | 叶 | 俏 | 统 | 峭 | 瀑 |
| 跳 | 院 | 殊 | 驮 | 腮 | 吻 |
| 洗 | 眨 | 拴 | 吻 | 绍 | 胁 |
| 猪 | 终 | 坦 | 绣 | 填 | 锈 |
| 组 | 捉 | 渣 | 训 | 侠 | 帐 |

Note. Large, large neighborhood size; Small, small neighborhood size.
